# The effects of age on associations between markers of HIV progression and markers of metabolic function including albumin, haemoglobin and lipid concentrations

**DOI:** 10.1111/hiv.12103

**Published:** 2013-11-19

**Authors:** M Samuel, S Jose, A Winston, M Nelson, M Johnson, D Chadwick, M Fisher, C Leen, M Gompels, R Gilson, FA Post, P Hay, CA Sabin, for the UK Collaborative HIV Cohort Study (UK CHIC)

**Affiliations:** 1 Guy's and St Thomas' NHS Foundation TrustLondon, UK; 2 Research Department of Infection and Population Health, University College London, Royal Free CampusLondon, UK; 3 Department of Medicine, Faculty of Medicine, Imperial College London, St Mary's Hospital CampusLondon, UK; 4 Chelsea and Westminster Hospital NHS Foundation TrustLondon, UK; 5 Royal Free London NHS Foundation TrustLondon, UK; 6 South Tees Hospitals NHS Foundation TrustMiddlesbrough, UK; 7 Brighton and Sussex University HospitalsBrighton, UK; 8 University of Edinburgh, Western General HospitalEdinburgh, UK; 9 North Bristol NHS TrustBristol, UK; 10 The Mortimer Market Centre, University College London and Central and North West London NHS Foundation TrustLondon, UK; 11 King's College London and King's College HospitalLondon, UK; 12 St George's University Hospital, St George's Healthcare NHS TrustLondon, UK

**Keywords:** ageing, albumin, haemoglobin, HIV, lipid metabolism

## Abstract

**Objectives:**

We investigated whether age modified associations between markers of HIV progression, CD4 T lymphocyte count and HIV RNA viral load (VL), and the following markers of metabolic function: albumin, haemoglobin, high-density lipoprotein cholesterol (HDL-C), low-density lipoprotein cholesterol (LDL-C) and total cholesterol (TC).

**Methods:**

A retrospective analysis of data from the United Kingdom Collaborative HIV Cohort was carried out. Analyses were limited to antiretroviral-naïve subjects to focus on the impact of HIV disease itself. A total of 16670 subjects were included in the analysis. Multilevel linear regression models assessed associations between CD4 count/VL and each of the outcomes. Statistical tests for interactions assessed whether associations differed among age groups.

**Results:**

After adjustment for gender and ethnicity, there was evidence that lower CD4 count and higher VL were associated with lower TC, LDL-C, haemoglobin and albumin concentrations but higher triglyceride concentrations. Age modified associations between CD4 count and albumin (*P* < 0.001) and haemoglobin (*P* = 0.001), but not between CD4 count and HDL-C, LDL-C and TC, or VL and any outcome. Among participants aged < 30, 30–50 and > 50 years, a 50 cells/μL lower CD4 count correlated with a 2.4 [95% confidence interval (CI) 1.7–3.0], 3.6 (95% CI 3.2–4.0) and 5.1 (95% CI 4.0–6.1) g/L lower haemoglobin concentration and a 0.09 (95% CI 0.07–0.11), 0.12 (95% CI 0.11–0.13) and 0.16 (95% CI 0.13–0.19) g/L lower albumin concentration, respectively.

**Conclusions:**

We present evidence that age modifies associations between CD4 count and plasma albumin and haemoglobin levels. A given reduction in CD4 count was associated with a greater reduction in haemoglobin and albumin concentrations among older people living with HIV. These findings increase our understanding of how the metabolic impact of HIV is influenced by age.

## Introduction

The number and proportion of older people living with HIV infection are increasing. It is predicted that by 2015 half of the people living with HIV in the USA will be aged 50 years or above 1. In the UK, the proportion of people living with HIV who are aged > 50 years increased from 12% in 2002 to 22% in 2011, a phenomenon that is thought to be associated with both increased survival and increasing incidence of new infections among older individuals 2. In the setting of effective antiretroviral therapy (ART), non-AIDS-related disease has become the predominant cause of mortality among people living with HIV 3. HIV disease progression, as indicated by a reduced CD4 T lymphocyte cell count and increased HIV RNA viral load (VL), is associated with derangement in some markers of metabolic function, specifically lower plasma albumin concentration 4, lower haemoglobin concentration 5, and higher plasma triglyceride but lower plasma total cholesterol (TC) and high-density lipoprotein cholesterol (HDL-C) levels 6. While older age is associated with an increased risk of many non-AIDS-related causes of morbidity and mortality, independently of HIV infection, there are no data describing whether the impact of HIV disease progression on markers of metabolic function is greater among older people living with HIV compared with their younger peers.

There is evidence that age modifies the impact of HIV disease on immunological function. For instance, CD4 cell count declines are more rapid following HIV infection among older people living with HIV disease 7 and older age is also associated with an increased risk of AIDS-related morbidity after adjusting for the CD4 count 8.

CD4 cell count and HIV VL are used by clinicians to indicate the stage of HIV infection and guide ART decisions. Understanding how infectious and noninfectious consequences of HIV infection at a given CD4 count/HIV VL differ between older and younger people living with HIV may influence the interpretation of these markers in different age groups. In this paper, we investigate whether age modifies associations between markers of HIV disease progression (CD4 count/HIV VL) and plasma albumin, haemoglobin and plasma lipid concentrations.

## Methods

Data from the United Kingdom Collaborative HIV Cohort (UK CHIC) Study were used in this analysis. The UK CHIC Study is an observational cohort study of HIV-infected individuals aged over 16 years attending some of the largest HIV clinical centres in the UK from January 1996 onwards 9. All ART-naïve subjects with at least one visit between 1996 and 2011 were included in the analysis. Analyses were limited to ART-naïve individuals to focus on the impact of HIV disease, rather than ART-associated derangements, on the outcomes being assessed. To allow for possible miscoding of ART status, apparently ART-naïve individuals with an HIV VL of < 1000 HIV-1 RNA copies/mL were assumed to be receiving ART and were excluded from analyses. Those with a CD4 count > 1000 cells/μL were excluded for the same reason.

Data were analysed in stata v12 (StataCorp LP, College Station, Texas, USA). Multilevel mixed effects linear regression models were used to assess the associations between markers of HIV progression (CD4 count/HIV VL) and the following outcomes: haemoglobin, albumin, low-density lipoprotein cholesterol (LDL-C), HDL-C, TC and triglycerides. Data on whether plasma lipid concentrations were fasting or nonfasting were not available. For each outcome measure, we identified measures of the exposure of interest within a 90-day window. Each resulting exposure/outcome ‘pair’ was included in the analysis as a separate observation. Where a single exposure measure was paired to multiple measures of the outcome, all resulting pairs were included in analysis. Random intercepts were introduced into the models at the participant level (level 3) and at the outcome level (level 2) to account for multiple observations from each participant and multiple measures of outcome mapped to a single exposure. Each model included the main effect of age (stratified as < 30, 30–50 and > 50 years), CD4 count and HIV VL and included adjustment for gender and ethnicity. To investigate whether age modified the associations between CD4 count/HIV VL and each outcome, analyses were performed separately in each age group, and formal tests of interaction were used to assess the significance of any differences in the associations among age groups.

The UK CHIC Study was approved by a multicentre research ethics committee and by local ethics committees.

## Results

A total of 16670 subjects (78.9% male) contributed 38261 observations to the overall cohort. At the time of entry into the cohort, 23.1% were aged < 30 years, 68.7% were aged 30–50 years and 8.2% were > 50 years of age. The majority of participants self-classified as being of White (59.7%), Black African (20.5%) or Black ‘other’ (5.3%) ethnicity, with the remaining patients self-identifying as being of South Asian (0.8%) or ‘other’ (13.8%) ethnicity. The median (interquartile range) CD4 count and HIV VL at the time of entry into the cohort were 320 (180–487) cells/μl and 4.58 (3.81–5.17) log_10_ copies/mL, respectively. The median (interquartile range) concentration of each outcome was: albumin, 39 (33.0–44.0) g/L; haemoglobin, 127 (106–142) g/L; TC, 4.0 (3.5–4.9) mmol/L; LDL-C, 2.1 (2.0–3.0) mmol/L; HDL-C, 1.0 (0.9–1.3) mmol/L; and triglyceride, 1.3 (1.0–2.0) mmol/L.

### Associations with metabolic markers in the unstratified analysis

After adjustment for gender and ethnicity, there was evidence that lower CD4 count and higher HIV VL were associated with lower TC, LDL-C, haemoglobin and albumin concentrations but higher triglyceride concentrations (Table 1). Older age was associated with lower haemoglobin and plasma albumin and HDL-C concentrations, but higher TC, LDL-C and triglyceride concentrations.

**Table 1:** Associations between CD4 T lymphocyte count and HIV RNA load (HIV VL) and metabolic markers among antiretroviral-naïve participants

	Metabolic marker												
	Albumin (g/L)		Haemoglobin (g/dL)		Cholesterol (mmol/L)		LDL cholesterol (mmol/L)		HDL cholesterol (mmol/L)		Triglyceride (mmol/L)		
	β* (95% CI)	*P*†	β* (95% CI)	*P*†	β* (95% CI)	*P*†	β* (95% CI)	*P*†	β* (95% CI)	*P*†	β* (95% CI)	*P*†	
*n* (participants)	18656		14579		7895		3937		4923		7640		
*n* (observations)	26115		31255		10265		4896		6467		9616		
CD4 count (50 cells/μL)	0.34	< 0.001	0.12	< 0.001	0.02	< 0.001	0.02	< 0.001	0.03	< 0.001	−0.01	0.01	
	(0.31, 0.38)		(0.11, 0.12)		(0.02, 0.03)		(0.01, 0.03)		(0.02, 0.05)		(−0.02, −0.002)		
HIV VL (log_10_ copies/ml)	−0.45	< 0.001	−0.29	< 0.001	−0.21	< 0.001	−0.05	< 0.001	0.04	0.21	0.06	0.02	
	(−0.52, −0.37)		(−0.34, −0.24)		(−0.26, −0.17)		(−0.07, −0.03)		(−0.02, 0.10)		(0.01, 0.11)		
Age	< 30 years	1	< 0.001	1	< 0.001	1	< 0.001	1	< 0.001	1	0.03	1	< 0.001
	30–50 years	−1.06		−0.17		0.32		0.25		−0.06		0.26	
		(−1.39, −0.74)		(−0.26, −0.08)		(0.24, 0.40)		(0.15, 0.34)		(−0.17, 0.05)		(0.17,0.35)	
	> 50 years	−3.40	−0.61	0.41	0.35	−0.23	0.27	
		(−3.93, −2.88)		(−0.75, −0.46)		(0.29, 0.53)		(0.20, 0.49)		(−0.40, −0.06)		(0.13, 0.40)	

CI, confidence interval.

* β represents the estimated impact of a 50 cells/μL increase in CD4 count, a 1 log_10_ increase in HIV VL or a specified increase in age on the specified metabolic marker. All estimates are additionally adjusted for gender and ethnicity.

† *P*-values from likelihood ratio tests.

### Effect modification of associations by age

**Fig 1:**
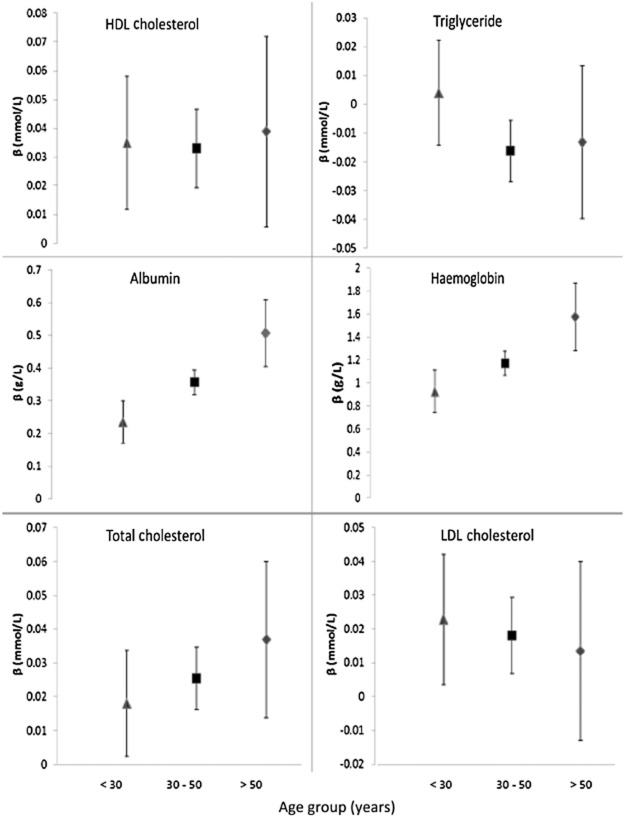
Age-stratum-specific estimates of associations between CD4 T lymphocyte count and albumin, haemoglobin, triglyceride, total cholesterol (TC), high-density lipoprotein cholesterol (HDL-C) and low-density lipoprotein cholesterol (LDL-C) concentrations. β represents the predicted effect of a 50 cells/μL increase in CD4 count on each outcome from a multilevel linear regression model adjusted for gender and ethnicity. 95% confidence intervals of estimates are also displayed. Separate estimates are given for participants aged < 30 years (Δ), 30–50 years (□) and > 50 years (◊).

In the adjusted analysis, there was evidence that age modified the associations between CD4 count and haemoglobin (interaction *P* = 0.001) and between CD4 count and albumin (interaction *P* < 0.001; Fig. 1). In particular, the estimated impact of a 50 cells/μL lower CD4 count on haemoglobin was a reduction of 2.4 [95% confidence interval (CI) 1.7–3.0], 3.6 (95% CI 3.2–4.0) and 5.1 (95% CI 4.0–6.1) g/L in those aged < 30, 30–50 and > 50 years, respectively. The estimated impact of a 50 cells/μL lower CD4 count on albumin was a reduction of 0.09 (95% CI 0.07–0.11), 0.12 (95% CI 0.11–0.13) and 0.16 (95% CI 0.13–0.19) g/L in those aged < 30, 30–50 and > 50 years, respectively. There was no evidence in the adjusted analysis that age modified the association between CD4 count and TC (*P* = 0.40), LDL-C (*P* = 0.84), HDL-C (*P* = 0.94) or triglyceride (*P* = 0.15) concentration, or the association between HIV VL and any of the markers of metabolic function.

## Discussion

Data from this large cohort study support the previously reported finding that deterioration in CD4 count and increasing HIV VL are associated with lower haemoglobin and albumin concentrations and greater derangement in plasma lipids. We are the first to present evidence that age modifies the association between the CD4 count and plasma albumin and haemoglobin levels; a given reduction in CD4 count was associated with a greater reduction in haemoglobin and albumin concentrations among older people living with HIV. Previously published work has suggested that haemoglobin and albumin are independent prognostic indicators in the presence of HIV infection 4,5.

Although we found evidence that worsening markers of HIV progression were associated with greater derangement in lipids, we did not find evidence that age modified these associations. One possible explanation for these negative findings could be a lack of power; our exploratory analyses support (nonsignificant) trends towards bigger increases in TC, smaller increases in LDL-C and greater decreases in triglyceride in the older age groups.

There is evidence that untreated HIV infection is associated with increased cardiovascular risk, but data regarding whether progressive deterioration in CD4 count increases cardiovascular risk remain inconsistent 10. The mechanisms by which HIV increases the risk of cardiovascular disease are debated. Our finding that HIV progression is associated with elevated plasma triglyceride concentrations would support the theory that HIV-associated triglyceridaemia plays an important role, although another study has suggested that the independent impact of triglyceride on cardiovascular risk among people living with HIV is small 11. It is also argued that HIV-associated cardiovascular risk may partially be mediated by HIV-specific pathways such as immune activation 12. Our study goes some way towards supporting this theory by providing evidence that markers of more advanced HIV disease were associated with a reduction in the plasma concentration of lipids such as LDL-C, a traditional risk factor for cardiovascular disease.

This study benefits from the large sample size of the UK CHIC Study. Limiting the analysis to ART-naïve patients also allowed us to assess the impact of the CD4 count and HIV VL independently of the impact of ART. There are limitations to this study, however. Data were analysed cross-sectionally, we therefore cannot assume a temporal association that changes in exposure preceded changes in outcome. We cannot exclude the possibility of unmeasured confounding as a result of a differential distribution of traditional risk factors for anaemia, hypoalbuminaemia or dyslipidaemia. Data were also lacking on the use of nonantiretroviral medications such as co-trimoxazole, which are more commonly prescribed among those with a low CD4 count and can be associated with adverse effects such as anaemia.

We did not adjust for recent opportunistic infection (OI) in this analysis, as OI was considered to be on the causal pathway between progressive HIV disease and the outcomes; it is possible that some of the apparent associations between progressive HIV disease and the measured outcomes were mediated through OI. Despite the large size of this cohort, there were fewer data for those aged over 50 years compared with the other age groups, limiting our ability to perform stratified analysis; there were also fewer data on plasma lipid profiles of this cohort than the other markers being assessed, raising the possibility that the study was under-powered to detect interactions with age when plasma lipid concentrations were the outcome assessed. Exclusion of participants with a CD4 count > 1000 cells/μL and/or a HIV VL < 1000 copies/mL reduced the risk that ART-experienced patients who may have been misclassified in the database were included in this analysis, but may have resulted in the exclusion of HIV elite controllers.

We complement the previously published finding that age modifies the impact of HIV disease on immunological function with evidence that age may also modify the impact of HIV on noninfectious pathology. CD4 counts are used by guidelines to guide ART decisions and by clinicians as a marker for the degree of HIV-associated disease that is likely to be seen. Our findings reinforce the importance of considering age in the interpretation of CD4 cell counts at both the individual and population levels. Further research to confirm these findings and to assess whether the changes in metabolic markers seen in this study are of clinical relevance is justified.

## Appendix

UK CHIC Steering Committee: Jonathan Ainsworth, Jane Anderson, Abdel Babiker, David Chadwick, Valerie Delpech, David Dunn, Martin Fisher, Brian Gazzard, Richard Gilson, Mark Gompels, Phillip Hay, Teresa Hill, Margaret Johnson, Stephen Kegg, Clifford Leen, Mark Nelson, Chloe Orkin, Adrian Palfreeman, Andrew Phillips, Deenan Pillay, Frank Post, Caroline Sabin (Principal Investigator), Memory Sachikonye, Achim Schwenk and John Walsh.

Central Coordination: UCL Research Department of Infection & Population Health, Royal Free Campus, London (Teresa Hill, Susie Huntington, Sophie Jose, Andrew Phillips, Caroline Sabin and Alicia Thornton); Medical Research Council Clinical Trials Unit (MRC CTU), London (David Dunn and Adam Glabay).

Participating Centres: Barts and The London NHS Trust, London (C. Orkin, N. Garrett, J. Lynch, J. Hand and C. de Souza); Brighton and Sussex University Hospitals NHS Trust, Brighton (M. Fisher, N. Perry, S. Tilbury and D. Churchill); Chelsea and Westminster Hospital NHS Trust, London (B. Gazzard, M. Nelson, M. Waxman, D. Asboe and S. Mandalia); Health Protection Agency – Centre for Infections London (HPA) (V. Delpech); Homerton University Hospital NHS Trust, London (J. Anderson and S. Munshi); King's College Hospital NHS Foundation Trust, London (H. Korat, M. Poulton, C. Taylor, Z. Gleisner and L. Campbell); Mortimer Market Centre, London (R. Gilson, N. Brima and I. Williams); North Middlesex University Hospital NHS Trust, London (A. Schwenk, J. Ainsworth, C. Wood and S. Miller); Royal Free NHS Trust and UCL Medical School, London (M. Johnson, M. Youle, F. Lampe, C. Smith, H. Grabowska, C. Chaloner and D. Puradiredja); St Mary's Hospital, London (J. Walsh, J. Weber, F. Ramzan, N. Mackie and A. Winston); The Lothian University Hospitals NHS Trust, Edinburgh (C. Leen and A. Wilson); North Bristol NHS Trust, Bristol (M. Gompels and S. Allan); University of Leicester NHS Trust, Leicester (A. Palfreeman and A. Moore); South Tees Hospitals NHS Foundation Trust, Middlesbrough (D. Chadwick and K. Wakeman); York Teaching Hospital NHS Foundation Trust (F. Martin and S. Douglas); St George's NHS Trust, London (P. Hay and M. Dhillon).
